# Metrics for maternity unit staffing in low resource settings: Scoping review and proposed core indicator

**DOI:** 10.3389/fgwh.2023.1028273

**Published:** 2023-03-15

**Authors:** William Stones, Anjali Nair

**Affiliations:** ^1^ Centre for Reproductive Health, Kamuzu University of Health Sciences, Blantyre, Malawi; ^2^ Heilbrunn Department of Population and Family Health, Columbia University Mailman School of Public Health, New York, NY, United States

**Keywords:** childbirth, human resources for health, midwifery, staffing adequacy, EMONC, facility

## Abstract

**Background:**

The lack of usable indicators and benchmarks for staffing of maternity units in health facilities has constrained planning and effective program implementation for emergency obstetric and newborn care (EmONC) globally.

**Objectives:**

To identify potential indicator(s) and benchmarks for EmONC facility staffing that might be applicable in low resource settings, we undertook a scoping review before proceeding to develop a proposed set of indicators.

**Eligibility criteria:**

Population: women attending health facilities for care around the time of delivery and their newborns. Concept: reports of mandated norms or actual staffing levels in health facilities.

**Context:**

studies conducted in healthcare facilities of any type that undertake delivery and newborn care and those from any geographic setting in both public and private sector facilities.

**Sources of evidence and charting:**

Searches were limited to material published since 2000 in English or French, using Pubmed and a purposive search of national Ministry of Health, non-governmental organization and UN agency websites for relevant documents. A template for data extraction was designed.

**Results:**

Data extraction was undertaken from 59 papers and reports including 29 descriptive journal articles, 17 national Ministry of Health documents, 5 Health Care Professional Association (HCPA) documents, two each of journal policy recommendation and comparative studies, one UN Agency document and 3 systematic reviews. Calculation or modelling of staffing ratios was based on delivery, admission or inpatient numbers in 34 reports, with 15 using facility designation as the basis for staffing norms. Other ratios were based on bed numbers or population metrics.

**Conclusions:**

Taken together, the findings point to a need for staffing norms for delivery and newborn care that reflect numbers and competencies of staff physically present on each shift. A Core indicator is proposed, “Monthly mean delivery unit staffing ratio” calculated as number of annual births/365/monthly average shift staff census.

## Introduction

The lack of usable indicators and benchmarks for adequate staffing of maternity units in health facilities has constrained planning and effective program implementation for emergency obstetric and newborn care (EmONC) globally. Many complications and deaths can be prevented by a set of medical interventions, coined “signal functions”, defined by the WHO, UNFPA, UNICEF, and leading maternal health institutions (1). These signal functions include (1) administering parenteral antibiotics, (2) administering uterotonic drugs, (3) administering parenteral anticonvulsants for pre-eclampsia/eclampsia, (4) manually removing the placenta, (5) removing retained products of conception using manual vacuum extraction or similar procedures, (6) performing assisted vaginal delivery (i.e., vacuum extraction or forceps delivery), (7) performing basic neonatal resuscitation, (8) performing surgery (CS delivery), and (9) administering blood transfusions.

Inadequate staffing can prevent EmONC facilities from providing both basic and comprehensive services. Staff in many settings around the world frequently report working under difficult conditions with large numbers of patients to attend, and suffer distress along with patients and families when complications or even fatalities arise as a result of inadequate staffing despite the best efforts of those on duty. Demoralization and stress can lead staff to seek redeployment to less challenging clinical areas, to the detriment of maternity and newborn services. Box 1 lists the particular challenges that arise from the service requirements of providing safe and acceptable delivery and newborn care, that render formulation of useful metrics difficult.

Box 1 **Special challenges for formulation of metrics for staffing of maternity and newborn services.**

**Figure 1:**
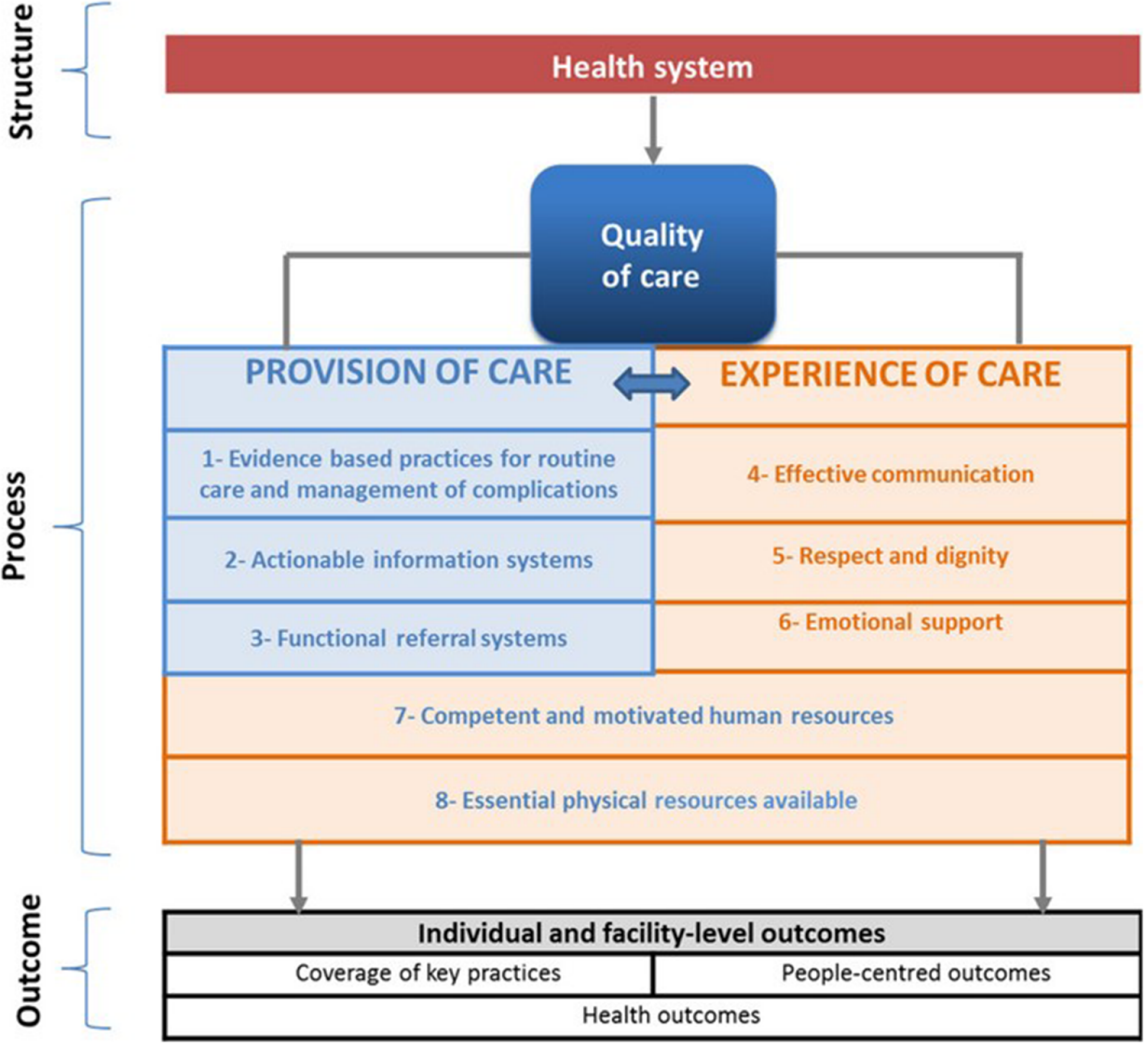
Reprinted from Tunçalp et al. 2015 (4) with permission.

– The need for health facilities to be able to assure 24-h cover, as large elements of maternity clinical presentations and care needs are unpredictable.
– Many life-threatening complications around the time of childbirth occur infrequently, so maintaining staff competence and confidence in dealing with them requires special planning, especially in facilities with a low annual number of births.
– To avoid ‘too much too soon, too little too late’ (2) in the provision of care, a high level of team capacity is needed, with competencies to identify and manage complications while having confidence to avoid unnecessary medicalization of uncomplicated labour, delivery and newborn care.
– As well as technical competence in the provision of care, maternity and newborn services need to be able to give consistent attention to the experience of care for women, their partners and families, in line with the Hulton and WHO frameworks for quality care (Figure 1) (3, 4). Humanistic, culturally appropriate woman-centred care is not an ‘optional extra’: apart from considerations of reproductive rights, adverse experiences of care are likely to deter women from accessing health facilities even in an emergency.

**Figure 2:**
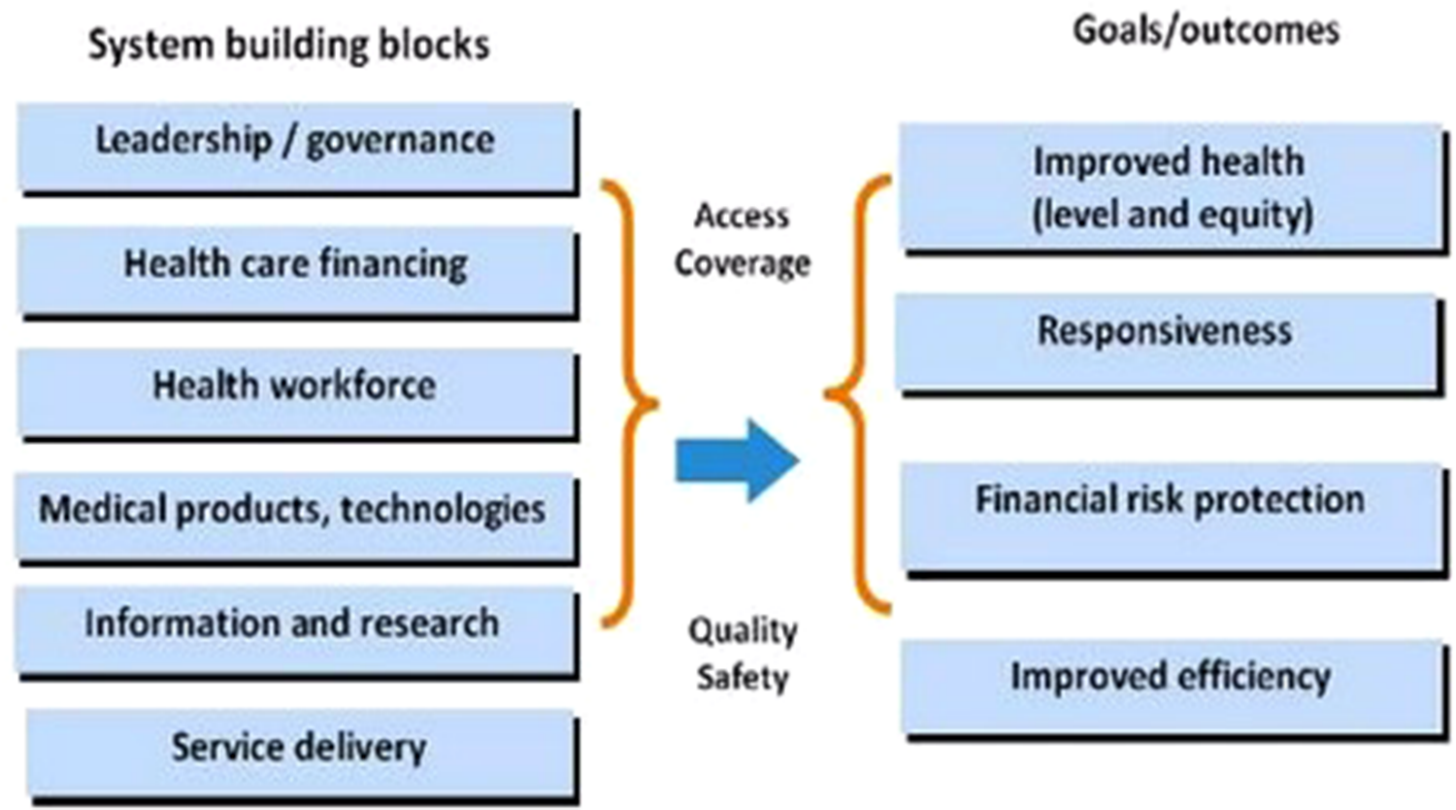
Reprinted from WHO 2007 (5) with permission.

The World Health Organization Health Systems framework recognizes the Health Workforce as a building block contributing to outcomes of improved health, responsiveness, risk protection and efficiency (Figure 2) (5).

In the context of maternity and newborn care the element of responsiveness is especially important and can be identified in four respects shown in Box 2.

Box 2 **Elements of Responsiveness required in maternity and newborn care services.**

– The capacity to recognise and respond to the personal and medical needs of individual women, newborns and families.
– Health facility staffing arrangements that allow for adjustment of staff allocation in response to peaks and troughs of demand that naturally occur on a daily basis.
– Ability to adjust staffing levels in response to longer term changes in utilization, for example an increase in delivery numbers at a particular facility, rather than restricting the staffing allocation to a fixed mandated complement based on the type of facility or other official norm.
– Recognition of case mix or acuity (population characteristics, general vs. referral facility, intensity of care needs) and skill mix (cadres/ professional groups, years of experience, special training and roles) in the planning of staff allocation.

The aspect of skill mix is partially addressed by recent moves away from the inclusive concept of “skilled birth attendant” towards recognition of the complexities of provider cadre and levels of training, experience and professional affiliation in different countries. WHO and the international health care professional associations have stated a preferred designation of “*skilled health personnel providing care during childbirth”* with an emphasis on competencies to *“provide and promote evidence-based, human-rights- based, quality, socioculturally sensitive and dignified care, to women and newborns; facilitate physiological processes during labour and delivery to ensure a clean and positive childbirth experience; and (iii) identify and manage or refer women and/or newborns with complications”.* (6). However, the current WHO intrapartum care recommendations do not address the matter of optimal staffing ratios and skill mix (7).

The landmark “State of the World's Midwifery” report (8) highlighted the challenge for human resource planning of the nominal facility headcount vs. full time equivalent (FTE^1^) staff actually present to provide delivery care. This means that metrics based on headcount of health care professionals against population size are an inadequate guide to this essential component of service availability.

The numbers needed and cadre mix of health care professionals involved in maternal and newborn care varies with both the population context of fertility and health in different countries, as well as by how the health system is organized and by national policies regarding levels of care and where women should give birth and want to give birth. Furthermore, emerging evidence continually adds to the list of effective interventions to improve maternal and newborn outcomes that can be provided by staff with midwifery and newborn care competencies, providing further layers of complexity and resulting in generally higher estimates of staffing needs than those reported in the 2014 report (9). These interventions may impinge in different ways across the continuum of care. For example, countries with a high burden of HIV infection require staff with relevant competencies for prevention of maternal to child transmission and sufficient time to provide the related components of care. However, when women have been stabilised on antiretroviral therapy with undetectable viral loads there is no excess care need during labor and delivery itself. By contrast, rolling out of measures to detect and manage fetal bradycardia in the second stage of labor such as use of hand-held Doppler devices and competencies in vacuum delivery have excellent scope to reduce intrapartum stillbirth and neonatal asphyxia but do require additional skilled staff presence in the delivery room (10). When a staffing model has already taken these components of care into account there is greater resilience should an emergency occur. Very recently, a formal trial has provided evidence that the presence of a second midwife in the delivery room can result in a clinically important reduction in perineal and sphincter injury during childbirth (11).

With regard to sick newborns, similar challenges regarding case mix/acuity and skill mix arise to those in labor and delivery care. Rogowski and colleagues analysed patient acuity and staffing arrangements in US newborn intensive care units (12). There were statistical associations between acuity and staffing levels, but nurse education, experience, and specialty certification were not reflected in nurse-to-infant ratios. The challenges of newborn unit staffing in the limited-resource setting of Kenya have been described (13). In the English National Health Service, a concept of “Qualified in Speciality” (QIS) is incorporated into staffing calculations, with for example a minimum of 70% of registered nurses needing to be QIS in lower acuity neonatal special care but all QIS for intensive care (14).

The detrimental impact of staff rotation has received attention in maternal and newborn health policy discussions, for example the Newborn Toolkit (15) where a recommendation is made for “*Non rotation of staff in order to ensure the development of institutional memory, knowledge and skills transference, continuity of care and the growth of an experienced workforce.”* The FIGO recommendations on staffing requirements noted that rotation can be problematic especially in countries that have followed a “nurse-midwife” professional model such that those working as midwives in delivery units might rotate to unrelated clinical areas (16). There may be staff preference for access to rotation, either for professional development or to take advantage of opportunities such as extra shifts that may be available. Rotation within maternity and newborn units may carry benefits to the service: for example, enabling staff mainly working in antenatal care to refresh their labor ward skills from time to time. This might ensure that advice given in antenatal services is current and consistent with actual practice in the labor ward.

Health facilities providing maternity and newborn care vary considerably in size around the world, from birthing centers with fewer than 300 deliveries per annum up to very large referral hospitals with as many as 30,000 annual births. There has been concern about the ability to offer safe care in small units, as they may fall below a critical mass that can provide all the needed elements of care. For example, 24 h cover with an on-site anesthesiologist or neonatal paediatrician may be impractical. Overall, in smaller hospitals units that maintain a 24-hour capacity to offer delivery care, staffing costs are higher than in larger units owing to loss of economies of scale in rota arrangements (17). Evidence is unclear as to whether maternal and newborn outcomes are necessarily worse in smaller relative to larger units: in a large United States analysis maternal outcomes were similar (18) whereas the adjusted odds of receiving guideline based neonatal sepsis treatment in Nepal were substantially greater in large hospitals (19). Smaller units are likely to have medical staff on-call from home rather than resident in the hospital: a study of low-risk births in Finland showed a greater risk of intrapartum stillbirth when the on-call arrangement was from home (20). A potential indicator and related benchmarks for maternity and newborn unit staffing needs to reflect expected variances related to unit size and cover arrangements.

Although the importance of staffing arrangements has been widely recognized in policy documents, a systematic review of human resources for health interventions for improving maternal health outcomes did not identify any studies, despite including “*Personnel systems: workforce planning (including staffing norms), recruitment, hiring, and deployment”* in the review scope (21).

With the aim of identifying potential indicator(s) and benchmarks for EmONC facility staffing that might be applicable in low resource settings, this report used a Scoping Review methodology to identify current global literature that may have advanced the field.

The objective of this review was to identify indicators and benchmarks for maternity unit staffing for delivery and newborn care, on which international consensus is currently lacking. The review aimed to consider size of health facility (number of births), case mix (risk status of clients/ patients), skill mix of staff (professional cadre, midwifery competencies, additional clinical roles in supporting maternity care), service organizational arrangements (rotas, rotation) and recognized overall health system resource contexts (low, middle and high income country settings). The review sought to identify any studies that linked staffing levels with maternal and perinatal outcomes. The focus was on reports that could be applicable in low resource settings whether generated in high or low resource countries.

## Methods

Table 1 shows the inclusion criteria related to the objectives in terms of Population, Concept and Context (PCC).

**Table 1:** Scoping review inclusion criteria related to the objectives.

Topic	What indicators and benchmarks for clinical staffing of maternity unit delivery and newborn care services are available?
Objective	The objective of this review is to identify indicators and benchmarks for maternity unit staffing for delivery and newborn care, on which international consensus is currently lacking. The review will consider size of health facility (number of births), case mix (risk status of clients/ patients), skill mix of staff (professional cadre, midwifery competencies, additional clinical roles in supporting maternity care), service organizational arrangements (rotas, rotation) and will recognize overall health system resource contexts (low, middle and high income country settings). The review will seek to identify any studies that link staffing levels with maternal and perinatal outcomes.
**Inclusion criteria: PCC**	
Population	Women attending health facilities for care around the time of delivery and their newborns (includes women and neonates referred in to health facilities after delivery for emergency care) (note that the scope does not include prenatal/ antenatal care or postnatal care of mothers and newborns beyond immediate/ emergency settings)
Concept	Reports of mandated norms or actual staffing levels in health facilities. Specific data to be extracted from the included studies will include: – Service elements (delivery care, newborn care) – Facility size (number of births – Health system level (primary, District level, Referral) – Numbers in professional cadres (Obstetricians, paediatricians, medical officers, clinical officers/ non physician clinicians, nurses, nurse/midwives, midwives, neonatal nurse practitioners, nursing/ maternity care/ newborn care assistants) – Rota type in operation (shifts per 24 h, day/night/weekend arrangements) – Rotation of staff to non-EmONC clinical roles – Rotation of staff within maternal/ newborn care roles (eg from delivery unit to antenatal/ postnatal wards) Maternal and perinatal outcomes (facility maternal deaths or near miss morbidities, perinatal mortality, admissions to neonatal nursery/ intensive care)
Context	The current scoping review will consider studies that have been conducted in healthcare facilities of any type that undertake delivery and newborn care, including BEmONC and CEmONC. Studies from any geographic setting will be eligible for inclusion as will those relating to both public and private sector facilities. Searches will be limited to material published since 2000 and material that is in English or French or at least includes an English language abstract.
Study types	Reports of Ministries of Health, Health Care Professional Associations, reports of analyses of service provision (eg DHS SPA series, SARA surveys), observational and quasi-experimental studies, formal comparative studies, modelling studies.
Search strategy	Syntax for online searching will be applied in Pubmed. Titles will be screened for potential relevance before scrutiny of abstracts where available for further sifting. Material identified at this stage will be read for data extraction using a template. Further searching of reference lists of reports will be used to identify sources not found in electronic searches, together with purposive searching of national Ministry of Health, non-governmental organization and UN agency website documents.
Data extraction	A rapid screen of titles will identify those that may contain mandated norms or actual staffing levels for hospital delivery and newborn care facilities (EmONC). Data will be extracted to a table by one investigator for this review.

Reference was made to the Prisma-ScR Checklist (22) as far as possible within the limitations of the present study. Owing to time constraints the investigators undertook data extraction individually rather than a model of two independent investigators that would have been ideal.

Search syntax in Pubmed was as follows:

(“maternal” OR “newborns” “neonates” OR “neonatal” OR “childbirth” OR “maternity” OR “obstetric”) AND (“staffing” OR “staff allocation” OR “human resources” OR “full-time equivalent employment” OR “staffing adequacy” OR “provider ratio”)

A second search added.

AND (“indicator” OR “recommendation” OR “benchmark”) to the above syntax.

Manual online searching of Ministry of Health and program websites in low resource countries for national maternal and newborn health service policy documents available in English or French was also undertaken.

## Title screening

Pubmed search output files (.nbib) were uploaded into a reference manager and titles were flagged for further screening. Downloads of Ministry of Health reports and grey literature were screened by content, aiming for inclusiveness where only a single report was available for a particular country and recognizing that titles alone might not be a good guide to the content of interest for the review.

Titles retained for further scrutiny from the search output files were as follows:

The initial Pubmed search generated 1,204 titles of which 170 were retained after initial screening. Titles retained included 8 systematic or scoping reviews. The second Pubmed search identified 42 titles of which 9 were retained: these proved all to have been identified in the initial search.

From reports and grey literature, 39 reports were identified of which 20 were retained for further examination after initial scrutiny (Table 2).

**Table 2:** Scoping review flow chart.

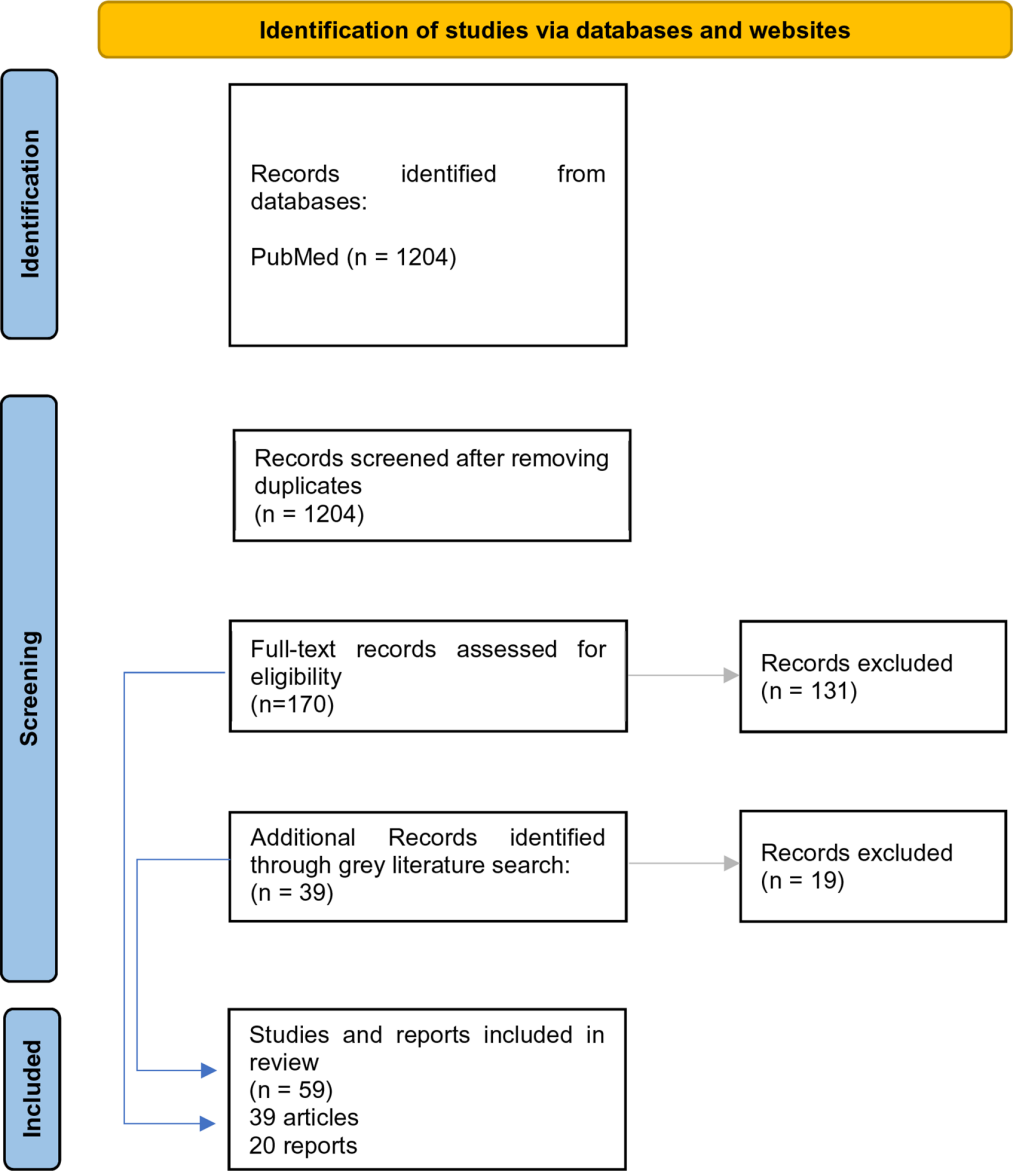

## Data extraction

Individual investigators undertook selection for data extraction using a template in Microsoft Excel, limited to the Abstract where access to the full paper or report was restricted but accessing the full paper or report where available. Data extraction was undertaken for a total of 59 sources, comprising 20 reports/grey literature, 36 journal papers and 3 systematic reviews. A formal quality grading of sources was not undertaken.

## Results

### Overview of findings

Findings from data extraction are presented in Table 3 in terms of the geographic source, the type of material, clinical focus within EmONC, facility type and how staffing ratios are calculated, followed by detail of the material and comment on applicability for indicator development. 59 papers and reports were included of which 6 were global in scope. Sources by World Health Organization regional office were AFRO: 17, Europe:12, SEARO: 11; Americas: 7, EMRO: 4 and Western Pacific: 2. There were 29 descriptive journal articles, 17 national Ministry of Health documents, 5 Health Care Professional Association (HCPA) documents, two each of journal policy recommendation and comparative studies, one UN Agency document and 3 systematic or scoping reviews. 34 sources related to both delivery and newborn care, 5 only to delivery care while 18 related only to newborn care. Two sources related only to post abortion care (PAC). A majority of sources related to multiple facility types (40 sources) with others relating to referral or University hospitals, District and rural hospitals or primary health centres. Calculation or modelling of staffing ratios was based on delivery, admission or inpatient numbers in 34 reports, with 15 using facility designation as the basis for staffing norms. Other ratios were based on bed numbers or population metrics.

**Table 3:** Data extraction table.

Identifier	Type of study or report	WHO Region	EmONC service elements	Facility type	Type of metric	Staffing description	Strengths and Limitations
Allen 2012	Journal article: descriptive study	Europe	Both delivery and newborn care	District Hospital	Delivery numbers	This paper both reports analysis of an actual year's data plus a modelling exercise. For the one year data, BR + workload factors are compared to actual staffing levels. There were 16.2 births per day with a standard deviation (SD) of 4.0. On 80% of days there were 11–22 deliveries, whereas 10% of days had fewer than 11 deliveries and 10% had more than 22 deliveries. BR + gave a workload of 8.2 midwives on duty at all times. The mean ratio of midwives to mothers was 1.3 (0.77 mothers per midwife). The authors give detailed analysis of BR + workload factors and response to overload such as planned CS. Re allowance for peaks, they note that a 15% margin is often used but is insufficient for labour wards. Calculations in the columns M and O are extrapolated from the paper for 3,000 births’	This paper has very useful practical insights on workforce planning, especially the need to plan for known variations in burden eg elective CS, but also have a mechanism to deal in a timely way with unplanned surges of activity.
AlSerouri 2012	Journal article: descriptive study	EMRO	Both delivery and newborn care	Multiple facility types	Population size	The paper compares staffing levels including medical and midwifery cadres in rural and urban governorates in Yemen in 2010. The 186 midwives in the three rural governorates represent 1.0 per 10,000 population, vs. 1.7 for Aden (the urban governorate). Respective ratios for obstetricians, anesthetists and pediatricians are 0.02:0.09, 0.02:0.2, 0.04:1.1 respectively.	The main thrust of the report is to highlight the huge deficiency in health care professional staff providing maternal and newborn care (this was before the current war).
Ansari 2015	Journal article: descriptive study	EMRO	Both delivery and newborn care	Multiple facility types	Admission numbers	This covers PAC services in a sample of health facilities across the range of types and sizes in Afghanistan	Highlights the importance of dedicated PAC training to ensure high percentage of competency/confidence among providers, not necessarily imparted through broader EmONC trainings.
AWHONN 2010	HCPA Report or Publication	Americas	Both delivery and newborn care	Multiple facility types	Not given	Detailed specifications for care needs throughout labor, delivery and newborn care including patients requiring operative delivery. Page 37 summarizes recommendations by stage of delivery and postnatal care.	Focuses on actual care needs per mother-baby dyad: the process of converting these care ratios into staffing for a whole maternity unit is not addressed.
Biswas 2018	Journal article: descriptive study	SEARO	Both delivery and newborn care	District Hospital	Not given	This is a study of staff presence by observation in Bangladesh. It shows substantial non-availability of general and specialist medical staff especially for evening and night shifts including non-response to emergency calls. Nursing/midwifery cadres are below expected levels and night shifts are also problematic especially for newborn care.	Highlights the Bangladesh scenario of 1:2 doctor:nurse ratio as opposed to the desired 3:1 reverse ratio (given in a citation of a WHO report). Puts forward their observation tool as a means to close staffing gaps especially in evening/night shifts but it is unclear how much could be resolved without a big overall increase in staffing levels.
Bolan 2021	Systematic or Scoping Review	Global	Newborn care only	Multiple facility types	Not given	Scoping review of human resource challenges to quality newborn care. Many themes identified, including the issue of staff rotation and its adverse effect on provision: “Many articles included in this review also cited rotation within the facility or transfer to another facility as a major concern. In a study of 6 health facilities in Ghana, many HWs interviewed were concerned about yearly rotation of neonatal staff, as this hampered quality of care given the loss of experienced staff and the time required to get new staff up to speed. Dewez et al. described that when nurses trained in India in neonatal care and were rotated to other wards they lost confidence and neonatal skills."	Shows the broad range of themes that need consideration beyond simple numbers or ratios when planning an effective HR model for newborn care, many of which are also relevant for maternal care.
Callaghan 2003	Journal article: descriptive study	Western Pacific	Newborn care only	University Hospital	Patient numbers	VLBW neonatal outcomes are analyzed against nurse staffing per shift ratios. Death was associated with an infant to staff ratio of 1:1.61 vs. 1.65 for those that survived. Adjusting for dependency care needs yielded a more complex pattern of survival, staffing ratios being apparently adverse in those with higher care needs.	Shows the complexity of trying to see an influence of staffing ratios amid the fine detail of NICU care provision. The authors advocate for observation based study.
Clark 2014	Journal article: descriptive study	Americas	Both delivery and newborn care	Multiple facility types	Patient numbers	110 hospitals in the USA contributed data to this study of staffing ratios during oxytocin augmentation of labor or induction. There were no clear statistical associations between the proportion achieving 1:1 staffing for these patients and adverse outcomes eg birth asphyxia	Specific to US practice of oxytocin administration.
Compaoré 2014	Journal article: descriptive study	AFRO	Both delivery and newborn care	Multiple facility types	Facility designation	The absolute minimum number of required staff in CEmONC facilities was pre defined as: three midwives (one each for morning, afternoon and night duty) to guarantee the presence of a skilled birth attendant twenty four hours a day, seven days a week in the maternities, and two surgical assistants and two anaesthesiologist assistants for providing 24/7 assistance to physicians with surgical skills. The median actual numbers at District hospitals were 3.9 midwives, 2.5 surgical assistants, 2.2 anesthesiology assistants, 1.6 physicians with surgical skills. At Regional hospitals the corresponding median numbers were: 9.3, 6,4, 4.8, 2.2.	The prior minimum staff complement appears to be extremely low, i.e. one midwife per shift at a CEmONC facility- even with this low bar the actual staffing was very low, and lower in district vs. regional hospitals. The analytical approach does not allow computation of ratios per delivery.
Compaoré 2022	Journal article: descriptive study	AFRO	PAC only	Multiple facility types	Facility designation	This report assesses PAC availability as a component of CEmONC in SSA health facilities Unfortunately there are no details of staffing arrangements and whether PAC is provided in general wards, labor wards or elsewhere. The issue of access to ICU/ specialist care for women with complications is addressed.	The paper is included here as it is of great value for policy and planning around PAC but not for the current purposes. The MCS-A could possibly be a source for further work if there is more detail on staffing arrangements
Corchia 2016	Journal article: descriptive study	Europe	Newborn care only	Referral Facility	Patient numbers	Staffing ratios for NICUs in Italy are estimated from monthly surveys. Hospitals had between 4 and 50 NICU beds and median daily number of patients 14.5, range 3.4 to 48.7. The number of nurses on duty per shift was a median 4.2, range (range 0.7–10.8) and the median nurse to patient ratio was 0.3 (range 0.1–0.9). In regression analyses adjusting for acuity scores the estimated mean daily numbers of nurses per shift for 10, 20 and 30 mean daily numbers of patients were 2.8 (1 nurse every 3.5 infants), 5.2 (1 nurse every 3.9 infants) and 6.9 (1 nurse every 4.3 infants).	This report of NICU highlights the variability of staffing levels in Italian units and emphasises the importance of a “professional goal oriented” rather than ‘bureacratic, task oriented’ approach to staffing planning with an emphasis on fostering a favorable professional environment. The data on activity vs. ratios could be useful for analysis of the ‘economy of scale’ in large units but is not fully developed in the paper.
Ethiopia1	Report: National MoH document	AFRO	Both delivery and newborn care	First level (eg Health Centre)	Facility designation	MCH services at health centres: minimum of 3 midwives and 5 nurses. Health Centre also has 2 Health Officers (NPC) and an ‘optional’ General Practitioner.	Limitations: does not specify detail of working arrangements for the nurses and midwives, eg shifts, leave cover, extent of responsibiltiy for non-EmONC clinical activities. Strength: specifies that MCH services shall be directed by a licensed ‘midwife nurse'
Ethiopia2	Report: National MoH document	AFRO	Both delivery and newborn care	District Hospital	Facility designation	Obstetrics and Gynecology services at General Hospitals: minimum for Delivery Services of 6 midwives. Ob/Gyn ward complement minimum of 4 Midwives and 10 Nurses. Further 3 midwives for outpatient services. OR services shared with Surgery but include anesthesiology and nurse specifications. Licensed Ob/Gyn available within 30 min, 24 h cover. Minimum of 2 Ob/Gyn specialists. Neonatal services at General Hospitals: nursing numbers not specified but to include “nurses with experience in neonatal care” as well as pediatrician and neonatologist.	Strengths: Service is directed by a licensed obstetrician and gynecologist with a minimum of two years' work experience; Neonatal services directed by a licensed pediatrician (no experience specified. While the staffing numbers are specified as a minimum based on designation, there is scope to adjust: “The number and type of technical staff shall be determined by the volume and type of work carried out (Workload Analysis)."
Ethiopia3	Report: National MoH document	AFRO	Both delivery and newborn care	Referral Facility	Facility designation	Speciality Centre for MCH (Draft document for public comment). Scope of services includes CEmONC plus major Gynecology surgery including oncology. Neonatal and Adult intensive care. Draft may be incomplete (see Limitations)	The draft may be incomplete as the staffing list is minimal with only 3 midwives and 8 nurses. also the description of ward areas does not match the scope of services, eg no postnatal or gynae ward.
Ghana1	Report: National MoH document	AFRO	Both delivery and newborn care	Multiple facility types	Admission numbers	This report used WHO's Workload Indicator for Staffing Needs statistical tools to provide total staffing norms based on Outpatient and Inpatient numbers but does not specify the clinical areas, so MNCH services and staffing numbers are not separately identified.	Not possible to use these data for MNCH-specific purposes.
Ghana2	Report: National MoH document	AFRO	Both delivery and newborn care	Multiple facility types	Admission numbers	As above (this is the technical report on which Ghana1 is based)	Not possible to use these data for MNCH-specific purposes.
Hamilton 2007	Journal article: descriptive study	Europe	Newborn care only	Referral Facility	Patient numbers	This is a one year neonatal intensive care staffing study in the UK, analyzed against a 1:1 ratio.. Overall median nursing provision ratio was 0.92 (mean, 0.96; SD 0.31) indicating that the average shift was understaffed. Median specialist nursing provision ratio was 1.3 (mean, 1.42; SD 0.78). There was a relative decrease in mortality with a higher specialist nursing ratio.	This study used a QIS approach to compute a ratio based on neonatal care-qualified staff. It only provides ratios to patients not QIS vs. non QIS proportion.
Hanson 2019	Journal article: descriptive study	SEARO	Newborn care only	Multiple facility types	Patient numbers	52 NICU were studied using records and observations. 6 and 8 nurses per 10 beds in public secondary hospitals and medical colleges, respectively was calculated as the staffing average.	The ratio is based on ‘per 10 beds’ which makes comparison difficult. The study cites the India Newborn Action Plan as the source for a benchmark but that document does not appear to contain a staffing benchmark.
India1	Report: National MoH document	SEARO	Delivery care only	Multiple facility types	Facility designation	Specifies minimum staffing for hospitals providing Basic and Advanced services, nursing complement includes one Head and two Nurses. Head of service specified as specialist ObGyn, “MBBS Doctors for round the clock service” but numbers of doctors not specified. Specifies a Pediatrician but this is covered in more detail in India3 below	Reflecting Indian health system arrangements the staffing model is weighted to medical rather than nursing/ midwifery cadres.
India2	Report: National MoH document	SEARO	Delivery care only	First level (eg Health Centre)	Facility designation	Includes delivery care and medical termination of pregnancy services although titled ‘Clinic’. One doctor, MBBS or senior for Clinic and DGO or senior for government licensed termination facilities. One nurse for Clinic and two for termination facilities.	Reflecting Indian health system arrangements the staffing model is weighted to medical rather than nursing/ midwifery cadres.
India3	Report: National MoH document	SEARO	Newborn care only	Multiple facility types	Facility designation	Specifies norms for two levels of service, one providing post-delivery Newborn care, prevention of Hypothermia, management of low birth weight baby, jaundice and the second level: Treatment of babies with respiratory failure, *c* pap, ventilator, seizure disorder, septicemia, jaundice, LBW and VLBW babies. Medical leadership is pediatric qualification with experience of 3 years ‘desirable’ for lower and ‘mandatory’ for higher level services. ‘MBBS doctors for round the clock cover’. Ophthalmology specialist for higher level services. Nursing: 1 per 5 beds in lower level, 1 per 3 beds in higher level general and 1 per 2 beds in NICU.	Reflecting Indian health system arrangements the staffing model is weighted to medical rather than nursing/ midwifery cadres. Strength: while mostly based on facility designation the nursing requirement is a ratio per bed number, though not specifying how this translates to shift arrangements.
India5	Report: National MoH document	SEARO	Both delivery and newborn care	Multiple facility types	Delivery numbers	Page 40 of this report has a table of staffing for sub-centers, primary health centers and district hospitals. The latter subdivides by delivery numbers. Specifies that “HR posted in the labor room should not be rotated outside the labor room.” Delivery numbers are assumed to be per month (not stated in the table but monthly numbers are used elsewhere in the report). Staff nurse numbers: 100–200 deliveries—4, 200–500–8, >500–10. Medical staff complement is also specified plus a complement of ANMs but it is stated that ‘all normal deliveries should be conducted by staff nurses’.	This is a realistic and service-focused plan that gives a good amount of detail. The rationale for the escalation of staff numbers in relation to delivery numbers is not given and may be too rigid, especially for very large units of more than 500 births per annum.
Jenkin-Cappiello 2000	Journal article: policy recommendation	Americas	Both delivery and newborn care	University Hospital	CHPPD metrics	This is a summary of a recommended model for calculation of staffing based on hours required for a range of maternity care tasks and culminating in a ratio. Actual total nursing care hours (TNCH) are divided by total patient required hours (TPRH) with the acceptable norm being 0.8–1.0.	The model is a local application and its wider generalizability is not clear.
Kaur 2019	Journal article: descriptive study	SEARO	Both delivery and newborn care	Multiple facility types	Per bed	Staffing is reported as part of a facility and user survey in Bihar, India. Staffing levels are reported as a percentage of mandated requirements, which are based on the number of beds at the facility. Auxiliary Nurse-Midwives are counted among health care professionals in this analysis. Staffing index was 69% (range: 11% to 100%) in PHCs, indicating 31% of health worker sanctioned positions, as reported by the MOIC, being unfilled. The average staffing index at PHCs was found to be the highest for the ANMs, indicating a high proportion of sanctioned positions being filled. However, the requirement of ANMs, as mandated by the IPHS guidelines, was fulfilled in only 42% of the PHCs. The mandate of having at least one MO at a PHC was fulfilled at all PHCs. However, the sanctioned positions varied and the average staffing index of available to sanctioned MOs was 70% for contractual (*n* = 129) and 68% for permanent staff. For District Hospitals the overall staffing index for three cadres was 55% (range 24% to 100%). The staffing index among the health personnel in DHs was found to be similar to PHCs; the staffing index was also highest for ANMs (78%) and lowest (35%) for laboratory technicians in DHs. For ANMs, the IPHS requirement of 0.45 staff per bed was fulfilled in only 15% of the DHs. The average staffing index for MOs was 52% and the requirement of essential MOs as per the IPHS guidelines was fulfilled in 53% of the DHs. Nearly 60% of the DHs had less than half of the sanctioned positions for MOs and nurses filled.	The calculations are against bed-wise norms hence hard to relate to actual delivery ward activity.
Kenya	Report: National MoH document	AFRO	Both delivery and newborn care	Multiple facility types	Facility designation	Uses Workload Indicator of Staffing Needs (WISN) methodology with category and individual allowance factors. Staff norms as follows for referral, county hospitals and health centres respectively: Medical Officers (clinical area not specified) 50,16,2; ObGyn specialist 3,2,0; Neonatologist 2,1,0; Anesthesiologist (all surgical areas) 6,2,0′ Clinical Officers in Reproductive Health 2,2,1; Clinical Officers in Pediatrics 6,2,1; Clinical Officers in Anesthetics (all surgical areas) 16,6,0; Registered Midwives 60,20,6, Pediatric Nurses 10,2,0 (theatre and anesthetic nurses specified separately)	This report has exhaustive detail of the different cadres contributing to health care including EmONC. The WISN methodology is used to generate national staffing needs estimates but does not provide the level of detail needed for EmONC-specific analysis. While Midwifery and Pediatric Nurse cadres are specified it is possible that other nursing cadres contribute to EmONC as Kenya has a predominantly nurse-midwife model. The Facility Type specification means that calculation of patient:staff ratios is difficult but could possibly be pursued using average admissions/ delivery numbers per facility type from DHIS.
Kokangul 2017	Journal article: modelling study	Global	Newborn care only	Referral Facility	Patient numbers	This is a modelling study for NICU staffing. The model results are hard to understand.	Included just for interest- very hard to understand.
Langenneger 2019	Journal article: policy recommendation	Global	Delivery care only	Multiple facility types	Facility designation	Blueprint/recommendations for central or tertiary hospitals in low- and middle-income countries. Recommended staff to patient ratios ≤ 14:1 for specialists, ≤1:2 for nurses, ≤1:5 for physiotherapist. Also recommended physiotherapists be available 7 days a week and nutritionists, psychologists, and occupational therapists be available during working hours	Model was used to develop and implement a way to develop and implement obsteric critical care unit at central hospital in South Africa, “can be implemented in central or tertiary- level hospitals in both low- and middle- income countries"
Malawi2	Report: National MoH document	AFRO	Both delivery and newborn care	Multiple facility types	Facility designation	Norms as follows for Central (referral) Hospitals, District Hospitals, Community/Rural Hospitals, Health Centres respectively: Medical Officers/ general doctors 6,5,1,0; ObGyn specialist 10,0,0,0 (but note that 1) there are also ObGyn Registrars at 4 per year of study, 4 year program and 2) Family Medicine graduates are expected to provide ObGyn and Neonatal cover at District hospital level, 2 per hospital); Neonatologist 1,0,0,0 (but note Central hospitals have 10 general pediatricians, 4 registrars per year of study and 6 Medical Officers who would contribute to newborn care); Anesthesiologist (all surgical areas) 5,0,0,0 (But note that Central hospitals are expected to also have 4 anesthetics registrars per year of study) Clinical Officers in Ob/Gyn 4,0,0,0; Clinical Officers in Pediatrics 4,0,0,0; Clinical Officers in Anesthetics (all surgical areas) 21,4,2,0; Nursing/ midwifery cadres are not broken down by clinical area in the hospitals in this report. Health Centres are mandated to have 14 nurses and 6 non physician clinicians with roles across all services.	This model shows an emphasis on deploying specialists at Central (referral) level and also makes extensive use of speciality trainees (Registrars) at that level. Family Medicine specialists along with Medical Officers and NCPs are expected to be the mainstay of medical provision at District level. This document refers to other Nursing Guidelines for details of nursing allocations (not seen).
Massad 2020	Journal article: descriptive study	EMRO	Newborn care only	Referral Facility	Patient numbers	The paper covers all facilities in the Palestinian territories and activity for 2016. Human resources included 11 neonatologists and 38 general pediatricians providing services in NICUs. 86/637 (13.5%) of nurses had specialized qualifications. Rates of training specifically on newborn resuscitation were low among all cadres.	The study highlights inequalities of distribution of neonatal services including staffing within the Palestinian territories.
Michel 2019	Journal article: descriptive study	Europe	Newborn care only	Referral Facility	Patient numbers	English abstract only used (text is German). Compares NICU staffing in Germany with other European countries. Quotes “significant lower nurse-staffing in Germany compared to the other countries (90.4 vs. 95.8%, *p* < 0.001). In addition, the average nurse-to-patient ratio was worse in German neonatal ICUs (3.0 vs. 2.3, *p* < 0.001). The presence of senior doctors is also lower in German neonatal and pediatric ICUs compared to the other countries (on weekdays: 12.0 vs. 14.6 h, *p* = 0.04; on weekends: 8.9 vs. 13.2 h, *p* = 0.003)".	Requires further scrutiny of the full manuscript. Are the percentages referencing a staffing norm?
Neogi 2011	Journal article: descriptive study	SEARO	Newborn care only	Referral Facility	Facility beds	Table 4 in the paper shows staffing ratios for doctors and nurses in 8 NICU in different states and territories of India. Ratios of nurses:beds ranged from 1:1.07 to 1:2 doctors:beds ranged from 1:2.6 to 1:7.0.	The authors analyzed differences in case fatality rate in the units under study. They note: “14% of the variation in the CFR could be explained by the number of nurses (r2 = 0.14, 95% CI −0.21, −0.49)” but medical staffing had no effect in their model.
Nepal	Report: National MoH document	SEARO	Both delivery and newborn care	Rural Hospital	Delivery numbers	"Annex 1.3a Functional Organogram” in this document mostly uses ratios as follows: Nurse patient ratio 1:6 in general ward, 1:4 in pediatric ward, 1:2 in high dependency or intermediate ward or postoperative ward) with one trained ward attendant per shift in each ward. Nurse / SBA Trained/ Midwife and mother ratio 1:2 in prelabor; 2:1 per delivery table and 1:6 in post-natal ward with at least one ASBA trained medical officer on duty and one office assistant are available in each shift. Newborn care/ NICU is part of the service configuration but staffing is not specified separately. Medical input to EmONC is not specified other than for PAC (see PAC column)	This report specifies staff to patient ratios for nurse/midwife staffing: if facility delivery numbers were known this would enable further calculations to be made. PAC is specifically mentioned but there is limited information about the scope or staffing of newborn care.
NIgeria3	Report: National MoH document	AFRO	Newborn care only	Multiple facility types	Facility designation	Mandated staffing for newborn care by facility level: Level 1 (Primary Health Centre)-"At least one skilled nurse has to be available round-the-clock for neonatal care per shift. One clinician, skilled in neonatal care, is required to oversee the clinical care “. Level 2 (specialty-level facility such as General and Cottage hospitals) “There should be at least two skilled nurses per shift and there should be an adequate number of clinicians to be able to do a ward round twice daily and to be on call.” Level 3 (Supspeciality newborn care) based on presence of neonatal pediatricians and “specialized neonatal nurses per shift with ratio of at least 1: 4 babies depending on level of severity and gestation"	Level of detail of staff skill mix and cover arrangements is limited
Ohnstad 2017	Journal article: descriptive study	Europe	Newborn care only	Multiple facility types	Delivery numbers	Recommended nurse-patients ratios- 0.33:1 for level 1 and 2 patients, 0.75:1 for level 3 patients, 1:1 for level 4 patients, 1.5:1 for level 5 patients	
Okonofua 2018	Journal article: descriptive study	AFRO	Both delivery and newborn care	Multiple facility types	Delivery numbers	Relates staffing levels to maternal mortality at 8 large Nigerian hospitals. The ratio of clients to providers ranges from 3 to 32, mean 15. The ratios appear to be based on total staff rather than per shift.	The data tables nicely capture different ways of combining medical and midwifery staff when computing ratios. They use ‘mProviders’ when combining all cadres. In-hospital maternal mortality in this report is shockingly high.
Olsen 2015	Journal article: descriptive study	AFRO	Both delivery and newborn care	Multiple facility types	Delivery numbers	Provides staffing ratios per facility but not disaggregated by clinical area within the maternity unit or by shift. Sample includes a high proportion of very small facilities (dispensaries). Workload ratio averages 27.2 deliveries per BEmONC staff.	The discussion section covers the issue of small facilities and advocates for shifting to larger facilities to maximise utilization of staff. Figure 1 in the paper shows cumulative number of deliveries per facility, showing that 72% of the facilities conduct fewer than 100 deliveries per year.
Oman	Report: National MoH document	EMRO	Both delivery and newborn care	Multiple facility types	Delivery numbers	This document gives national requirements for private health facilities. Nursing/midwifery staffing is based on ratios as follows: 2 midwives per 3 delivery rooms per shift (morning, evening, night), NICU 1:2 plus nurse in charge for each shift, operating theatre 3 per functioning theatre for morning and evening plus 3 on call at night. Medical staff requirement is not given in detail beyond a minimum of resident medical officer with ACLS training, access to a neonatal pediatrician and relevant specialists.	The ratio based approach for nursing/ midwifery cover is useful and recognises the need for full cover of delivery unit and SCBU at night.
Owens 2014	Journal article: descriptive study	AFRO	Both delivery and newborn care	Multiple facility types	Facility designation	Eval of 90 health centers and 10 hospitals in Southern Province of Zambia capacity to perform EmONC. 87 health centers and 10 hospitals had 24/7 service, 25 hc and 10 hospitals had ≥ 1 registered nurse, 29 hc and 9 hospitals had ≥ 1 clinical officer, 1 hc and 7 hospitals ≥ 1 assistant medical officer, 5 hc and 1 hospital had ≥ 1 generalist medical officer, 1 hc and 2 hospitals had ≥ 1 obstetrician.	
Patry 2013	Journal article: descriptive study	Europe	Newborn care only	University Hospital	Patient numbers	Ratios are given per shift over a 31 day period in a German NICU and compared with UK norms of 1:1 for NICU and 1:2 per high dependency patient. The calculated staff requirement was 12.10 ± 1.81 FTE per shift, higher than the actual 8.97 ± 0.87 FTE per shift available.	The calculation and reporting approach is easy to understand.
Pillay 2012	Journal article: descriptive study	Europe	Newborn care only	Referral facility	Admission numbers	England recommendations for neonate-nurse ratios are 1:1 for intensive care, 2:1 for high-dependency care, 4:1 for special care. In the SSBC network the recommendations are 1:2, 5:1, and 2.7:1, respectively. Media time spent per category of care was highest in intensive care. 54% of shifts did not meet standards, and nurses with a higher workload than recommended spent28% less median time on clinical care per baby.	
Rogowski 2015	Journal article: descriptive study	Americas	Newborn care only	Multiple facility types	Patient numbers	Nurse to patient ratio increased with level of infant acuity in both census and survey results. Survey: 0.34 for lvl 1, 0.38 for lvl 2, 0.49 for lvl 3, 0.66 for lvl 4, 0.95 for lvl 5. Census: 0.36, 0.41, 0.52, 0.72, 0.96 respectively.	Small units underrepresented, manget-accredited institutions and institutions with open heart surgery capacity overrepresented,
Sabde 2016	Journal article: descriptive study	SEARO	Both delivery and newborn care	Multiple facility types	Population metrics	Assesses EmONC in the context of the JSY demand side financing program in Madhya Pradesh, India. Uses the WHO2005 benchmark of 175 births per midwife per year (counting ANMs as midwives) assuming all births were delivered by this cadre. In different districts in CEmONC facilities the metric was 165 and 368, at ‘less than CEmONC’ facilities the metric was 169 and 201. At ‘less than BEmONC level the metrics for three districts were 117. 147 and 96.	Use of district wise births per midwife metric does not enable assessment of actual staffing of particular delivery units. DSF instruments such as JSY are very important given their huge scale in India and elsewhere and impact on staffing availability in detail would be highly relevant to potential impact on outcomes, but not using this methodology.
Sachan 2021	Journal article: descriptive study	SEARO	Both delivery and newborn care	Multiple facility types	Delivery numbers	52 facilities comprised of one medical college, one district hospital, seven CHCs, five PHCs and 38 sub-centres. They used staffing norms from the India 5 report cited above in this table. Against those norms, the percentages filled were: specialists 43%, medical officers 85%, staff nurse 42.9, ANM 40.8. Higher level hospitals had more filled staff nurse positions: 100% at the medical college, 75% at district hospital, 27.8% at CHC.	This study shows the relative ease of applying the India 5 model for staffing. It did not report detail that would have been useful, eg the rotation and shift arrangements in place and whether these met the specification in the standards.
Sandall 2014	Journal article: comparative study	Americas	Both delivery and newborn care	Multiple facility types	Delivery numbers	This is a major report analyzing outcomes in English maternity units in 2010–11 for more than half a million births. Staffing variables included: FTE doctors per 100 maternities; FTE midwives per 100 maternities; FTE support workers per 100 maternities; all staff per 100 maternities; Doctor-to-midwife ratio; Support worker-to-midwife ratio. There were 4.80 FTE staff for every 100 births of which 0.82 FTE were doctors, 3.08 FTE were midwives (1.11 to 4.71) and 0.90 FTE were support workers (0.05 to 2.88). Outcomes were assessed using “healthy mother and healthy baby” indicators and maternal/ perinatal mortality was not reported. Variation between women's characteristics had much bigger statistical effects than staffing variables. Levels of midwifery staffing were associated with only 2 of the 10 indicators, delivery with bodily integrity and intact perineum.	The large size of this data set collected under consistent reporting conditions in a publicly-mandated service makes fine detail of analysis possible. Interestingly the authors recommend that “The preferred measure to full-time equivalence for all staff groups would have been hours committed to maternal delivery care, or FTE for all groups over a whole pregnancy, birth and postnatal pathway”. For a service in a high income setting with very low mortality, the use of the ‘healthy mother, healthy baby’ construct is of interest.
Senegal	Report: National MoH document	AFRO	Both delivery and newborn care	Multiple facility types	Delivery numbers	This report specifies staffing levels for Senegal maternity facilities. It provides norms in terms of FTEs with increments for successive increases in delivery numbers.	The norms are specified in terms of FTEs which is advantageous relative to total head count. However, the translation into requirements per shift recognizing leave and absence is not made, thus facilities may be understaffed owing to absences even when compliant with the norm.
Sentilhes 2019	HCPA Report or Publication	Europe	Both delivery and newborn care	Multiple facility types	Delivery numbers	This is a consensus recommendation of the French HCPAs regarding maternity unit staffing, by size of unit. It specifies staffing norms in terms of effective coverage and also gives an “équivalent temps plein” ETP = FTE. Staffing of midwives is specified as 1:1 in labour rooms, with a base FTE of 6 midwives plus an additional FTE midwife for each additional 200 births. A unit of 2,500 births would have 13.5 FTE midwives. There are further tables for obstetric, neonatal and anesthesiology staffing requirements.	This is a clinically focused and realistic tabulation reflecting the French health system arrangements.
Sentilhes 2020	HCPA Report or Publication	Europe	Both delivery and newborn care	Multiple facility types	Delivery numbers	This is the English version of the above report. It gives more specifics on midwife ratios: 5.1 for 3,000 births and 7.2 for 4,500 births. A maternity unit's occupancy rate must not exceed 85%.	English version of the above
Sharan 2011	Journal article: descriptive study	AFRO	Both delivery and newborn care	Multiple facility types	Facility designation	Survey of all hospitals providing maternity care (national referral hospital, district hospitals, community hospitals), all health centers, random sample of 30% of health stations total sample: 118 facilities (18 hospitals, 47 health centers, 53 health stations), total needs and shortages recorded—For referral hospitals: needs 14 ob/gyn, 16 medical doctors, 7 anesthesiologist, 16 nurse-anesthetist, 20 nurse, 42 nurse-midwife, 35 health assistant/auxiliary nurse For community hospitals: needs 22 ob/gyn, 20 medical doctors, 6 anesthesiologist, 27 nurse-anesthetist, 68 nurse, 66 nurse-midwife, 70 health assistant/auxiliary nurse. For health center/station needs 252 ob/gyn, 260 medical doctors, 72 anesthesiologist, 220 nurse-anesthetist, 860 nurse, 572 nurse-midwife, 1,432 health assistant/auxiliary nurse	
Sherenian 2013	Systematic or Scoping Review	Global	Newborn care only	Referral Facility	Various	This systematic review of nursing ratios and outcomes in NICU included 7 studies (6 independent sources of data). It was not possible to undertake meta-analysis as the metrics used in each study were different. The authors conclude that “limitations of the existing literature prevent clear conclusions about optimal staffing strategies."	Main conclusion is the need for further research using consistent metrics to define staffing and outcomes.
Simpson 2019	Journal article: descriptive study	Americas	Both delivery and newborn care	Multiple facility types	Admission numbers	Hospitals with 500–999 births/yr had highest nurse-reported staffing compliance with AWHONN staffing guidelines. Largest hospitals (>2,500 births/yr) had lowest levels of adherence. Ratios were for nurse:patient ratios for specific care needs	Nurse-reported
Stones 2019	HCPA Report or Publication	Global	Delivery care only	Multiple facility types	Delivery numbers	This is a committee consensus recommendation (‘Expert Opinion’) from FIGO regarding delivery/ labour ward staffing. Minimum and Ideal recommendations are presented in tables, for (1) BEmONC without surgical care for delivery numbers between 1,000 and 3,000 per annum; (2) CEmONC facilities with between 1,000 and 7,000 births per annum, showing staff with and without surgical competencies; Numbers are not broken down by staff cadre.	These recommendations do not include newborn care beyond routine postnatal care, eg NICU or nursery admission. Anesthesiology also not considered. There are no recommendations for small maternity units (below 1,000 births per annum). Economies of scale achievable in larger units are reflected in range of ratios from 0.4 to 2.7 patients per staff in units from 1,000 to 7,000 births
Tanzania	Report: National MoH document	AFRO	Both delivery and newborn care	Multiple facility types	Delivery numbers	Norms are given for Dispensaries that may include delivery beds and for Health Centres but the allocation for EmONC is hard to compute. For District Hospitals the nursing allocation is: Nurse to Patient ratio 1:4 including nursing officers and assistant NOs. No specific allocation for NICU/ newborn care but Pediatric Ward is 1:8. No detail of medical cover for labor ward. Referral Hospitals: Labor ward 1:8 nursing ratio plus 1–2 specialists and 1–4 medical officers, also applies to Neonatal unit. There are additional tables for specific speciality/ referral hospitals.	Newborn care arrangements in non-referral hospitals are unclear.
Tawfik 2020	Journal article: descriptive study	Americas	Newborn care only	Multiple facility types	Admission numbers	Facilities categorized as “above-predicted” staffing associated with 21% lower odds of health care-associated infections in NICU ≥ 4%–6% lower odds for each NHPPD. Raw staffing associated with higher odds of death with each addition NHPPD (OR 1.07).	
Tucker 2002	Journal article: comparative study	Europe	Newborn care only	Referral Facility	Patient numbers	UK NICUs. There was no relation between absolute nurse-to-infant ratio for the whole cohort and mortality, but the ranked percentiles of nurse-infant ratio in every NICU showed that the odds of mortality rose as number of infants per nurse increased.	Main focus was on size of unit as a policy question.
Uganda	Report: National MoH document	AFRO	Both delivery and newborn care	Multiple facility types	Facility designation	Contains limited mention of staffing norms. Intensive care: minimum nurse: patient ratio of 1:1; “A qualified health worker is available 24 h a day, 7 days a week (A qualified health worker = nurse, midwife, Clinical Officer or Medical Officer).”; “There is a qualified health provider available at all times at the maternity. 7.24.1. A qualified health worker (midwife, CO or MO) is available 24 h a day, 7 days a week.” Reference is made to “Ministry of Public Service, Approved Staffing Norms, 2002”—document not seen.	The document is mostly headings of service categories and has limited detail.
Whr 2005	Report: UN agency document	Global	Both delivery and newborn care	Multiple facility types	Delivery numbers	The WHR contains an indicative recommendation based on an example District fertility pattern such that 3,600 births could be supported by 20 midwives, based on an expectation that each midwife would attend 175 births per annum. The latter estimate cites a 1991 report: https://apps.who.int/iris/bitstream/handle/10665/59744/WHO_SHS_CC_90.2.pdf?sequence=1&isAllowed=y In this report Annex 1 page 34 has 178 as the median number of births per midwife. The WHR proposes that 9–10 of these midwives would be stationed at the hospital. For the ratio column here, to adjust to 3,000 births a number of 8.3 hospital midwives is used. Shift arrangements are not specified but 2 is assumed for the calculation here. Issues of proportion of hospital birth, skill mix or case mix are not discussed.	These ratios have been cited as a global recommendation but in the original appear as more of an indicative approach to estimating numbers of staff needed.
WwBR+	HCPA Report or Publication	Europe	Delivery care only	Multiple facility types	Delivery numbers	The calculation includes all the activity of UK midwives including ANC, PNC and non-delivery care needs of maternity patients. It includes routine newborn care but not Special or NICU. The ratio calculation is based on hours of effort through the continuum, with adjustment for casemix in 5 groups of complexity, based on actual clinical data. The final ratio calculation is 29.5 births per wte midwife with a range of 27.3–31.5, and for hospitals with lower to higher proportions of complex cases ranges from 45 to 38 births per wte midwife.	Calculations are based on UK data. They reflect actual historical ratios so may be more or less favorable than ideal. The ratios are based on total midwifery workload for each pregnancy and birth, not just intrapartum care hence the ratios quoted here may be too low to apply to intrapartum care only.
Zbiri 2018	Journal article: descriptive study	Europe	Both delivery and newborn care	Multiple facility types	Delivery numbers	Study of public and private French maternity units of different sizes and staffing ratios. Main focus was the effect of staffing on CS rates but overall metrics are provided: FTEs for Obstetricians 0.49 per 100 deliveries, anesthesiologists 0.56, midwives 1.55	Intrapartum CS was related to lower obstetrician staffing, and elective CS to midwifery staffing.
Zhang 2021	Systematic or Scoping Review	Western Pacific	Both delivery and newborn care	Multiple facility types	Population metrics	This is a `systematic review of reports of educational level, cadre and roles of doctors, nurses and midwives in China, with a meta-analysis to derive population ratios and variances within those ratios.	This review notes the need to move towards ‘per birth’ calculations rather than ‘per population’.

### Metrics used in staffing analyses

A single study reported the staffing ratio metric of “Care hours per patient day” (CHPPD). Regarding the quality of staff in post or on duty in relation to their expected duties in maternal, newborn or postabortion care (Qualified in Service, QIS), most reports did not specify competencies beyond the primary clinical qualification. A single study of post abortion care included “confidence to perform post abortion care” as a measure of QIS, in this case reported as 68% (23). Also addressing QIS, a study of neonatal intensive care provision from the Palestinian territories reported that 86/637 (13.5%) of nurses had specialized qualifications (24). In a UK study, neonatal outcomes for very low birthweight babies were favourable where QIS as expressed by the ratio of nurses specialising in neonatology reached 1.3 (25).

Staff rotation arrangements were described in three sources: the Indian national policy document (26) specified that staff should not rotate from the maternity unit. The UK report of a staffing model implementation mentioned rotation within maternal and newborn services (27). Rotation of trained and experience staff was identified as problematic for quality newborn care in a scoping review (28).

### Staffing in relation to outcomes

A small number of studies related maternal outcomes to staffing ratios. Maternal mortality was reported in a study of 26,479 births in Nigeria with staffing ratios of 106:1 for doctors in post and 18:1 for midwives in post. There was considerable variation in the ratios between facilities studied, ranging from the most favourable at 1:1 to the most challenging at 22 births per provider. A statistical association was identified between adverse staffing ratios and the incident rate ratio for maternal death (29). It should be noted that staffing numbers here represent staff in post in a clinical department rather than FTE or actual ratios of staff on duty per shift in maternal and newborn care: much of the staff time is likely to have been allocated for a range of duties at the respective hospital such as antenatal clinic and ward duties. A large study aimed to examine birth outcomes in 2011 in England in relation to staffing levels: in this setting of very low mortality a composite outcome of “healthy mother and health baby” was used. There were 4.80 FTE staff for every 100 births (range 2.43–8.66, 20.8 births per provider FTE) of which 0.82 FTE were doctors, 3.08 FTE were midwives (range 1.11 to 4.71, 32.4 births per provider) and 0.90 FTE were support workers. Variation between women's characteristics had much bigger statistical effects than staffing variables in this analysis. Furthermore, levels of midwifery staffing were statistically associated with only two of the 10 indicators, delivery with bodily integrity and intact perineum. The FTE calculations reflect staff in post rather than shift-wise presence in the midwifery unit (30). The variation in staffing ratios within each of these two very different settings is noteworthy, perhaps especially in the English example given the relatively homogeneous structure of the National Health Service maternity system. Comparison of ratios of births per midwife, e.g. 18.1 in Nigeria and 32.4 in England, may be problematic owing to differences in the staffing estimates based on head count or FTE and the way in which the analysis apportions the contribution of a FTE midwife to hands-on delivery and newborn care.

Neonatal intensive care studies have identified a relationship between staffing levels and survival outcomes, for example *Callaghan* et al*. 2003* where an infant to staff ratio of 1:1.61 was reported for very low birthweight neonates who succumbed vs. 1.65 for those that survived (31). As noted above, in a UK study higher ratios of QIS staff were associated with improved newborn survival as was achieving a 1:1 staffing ratio per shift in a setting where many units were reporting ratios of 0.9 staff per neonate in the unit (25).

### Recommended and mandated staffing levels

Reports were identified in this review mandating staffing levels of maternity and newborn units authored by Ministries of Health, Health Care Professional Associations and the World Health Organization. The latter contains an indicative recommendation based on an example District fertility pattern such that 3,600 births could be supported by 20 midwives, based on an expectation that each midwife would attend 175 births per annum, derived from a 1991 report (32). Details of shift arrangements for delivery care and participation in other care activities are not given and the indicated number appears to be a head count rather than FTE or shift wise calculation.

FIGO provided a set of staffing recommendations based on delivery numbers at health facilities, recognizing that economies of scale can occur in larger units where dealing efficiently with peaks and troughs of demand may be easier to manage, as well as nurturing of a cadre of experienced staff able to take on a shift-leading role. The consensus statement also recognises the variation in care needs during a maternity episode, with ratios broken down by stage of admission, delivery and postnatal care, ranging from 1:8 in a latent phase observation area to 2 staff per 1 patient during active pushing in the second stage and for immediate postnatal observations (16). The model does not include the contributions of specialists including components of obstetric high care, anaesthesiology and neonatal paediatrics. The French health care professional associations including obstetrics, anaesthesiology, paediatrics, nursing and midwifery produced joint staffing recommendations for maternity units, specifying a 1:1 ratio in labour rooms, with a base FTE of 6 midwives plus an additional FTE midwife for each additional 200 births, meaning that a unit of 2,500 births would have 13.5 FTE midwives (33). This group further specifies that 5.1 midwives for 3,000 births and 7.2 for 4,500 births should be available per shift to provide unscheduled care, thus combining an element of FTE based analysis with consideration of numbers required on duty at any given time (34).

Ministry of Health documents frequently reported staffing norms according to facility designation. Notable reports specifying maternity unit staffing in relation to delivery numbers at the facility are those from India and Senegal. For India the allocation of medical and nursing/midwifery and support staff is specified in detail, for example for Staff Nurses (expected to conduct deliveries there would be 4 for a unit with 100–200 deliveries per month, eight for a unit with 200–500 deliveries and 10 for units delivering more than 500 mothers per month. The shift arrangements translating head count to FTE and staff actually present on shift is not specified (26). In Senegal, a similar incremental model is mandated with three FTE midwives specified for a unit with 31–60 monthly births, rising to 10 staff for a unit of 300 monthly births (35). Staff absence on leave, sickness or training is not factored into this model and detailed specifications for medical staff participation in maternal and newborn care is not included.

## Discussion

Taken together, a key observation from the material studied is the lack of consistency and clarity regarding the method of calculation of staffing norms or observed levels. Indeed, most sources do not provide a rationale or justification and do not specify whether the numbers reported are of staff in post, Full Time Equivalent (FTE) staff or staff physically present on the unit. This renders efforts to compare findings or norms across contexts very difficult and would also present difficulties for planners hoping to use these data to estimate hospital, District level and national staffing needs or benchmarks. Reported neonatal nurse staffing ratios most commonly reflect staff actually present on a shift, whereas reported numbers for maternity care are the least consistent in this regard.

The strongest staffing analyses and models seen in the review are those that are based on delivery or actual patient numbers rather than facility designation. In India there have been developments in specifying public health facility staffing norms in some detail, previously in terms of facility designation but the 2016 standards (26) add a refinement of additional staffing for each 200-birth increment. The French consensus document from the health care professional associations follows an approach based on unit size and activity and is notable for the detail provided regarding needs for obstetric, paediatric and anaesthesiology cover. The current Senegal reporting system is aligned with data capture into the District Health Information System (DHIS-2) and the technical document mandates staffing increments based on delivery numbers at the facility. A number of reports assess neonatal intensive care staffing needs in relation to admission numbers and acuity, predominantly from high income countries but including some from low- and middle-income settings. The matter of staff rotation is explicitly mentioned as undesirable in a number of sources. However, no studies describe or formally compare within-unit or external rotation practices in relation to patient outcomes or unit functionality.

The literature provides a strong sense of the importance of adequate staffing for EmONC with many studies identifying gaps in coverage, both in terms of numbers but also skill mix and competencies. The importance of contextualizing staffing arrangements for the local population and service needs is also emphasized and is consistent with a view that a single benchmark for staffing levels is likely to remain elusive. A critical issue is the way that staffing norms are expressed: while several reports recognize the need to capture workforce in terms of Full Time Equivalent (FTE) staff rather than numbers of staff nominally in post, there remains a gap between FTE and actual presence of staff on the delivery unit for a particular shift. For example, the Senegal model specifies a minimum of three FTE staff for facilities with low delivery numbers, but such a complement does not mean that one member of staff would be physically present for every shift once the hours of the working week, sickness absence and annual leave are taken into account. The FIGO consensus statement specifies recommended staffing in terms of FTE present on each shift.

## Implications for policy and programming

For maternal and newborn health planners to estimate staffing requirements, operationalize norms and benchmarks and undertake analytical studies exploring service models and their impact, consistent approaches to calculation of staffing ratios and their application as norms or benchmarks is needed. From the present review findings, the lack of clarity across the literature regarding staffing metrics is evident. Documents reviewed for this scoping exercise comprise a mixture of mandated and recommended norms. Often, mandated norms have arisen from previous reports of actual staffing levels that have not necessarily been shown to be ideal or desirable. An example is the World Health Report 2005 (32) where a workload of 175 births per midwife is recommended, based on 1990 experience in Rwanda in a District setting. This ratio has been heavily cited as a WHO-mandated norm, perhaps beyond the intentions of the originators.

While it appears unrealistic to anticipate that an authoritative globally applicable benchmark for delivery unit and newborn care staffing can be identified from existing literature, for policy and programming an interim solution is needed so that indicator(s) are available that governments (at national and sub-national levels) and facility directors find useful for planning and implementing high-quality maternal and neonatal health services. Such indicators need to accurately measure the actual situation in the maternity ward, and offer the ability to relate these to the outcome of interest especially delivery of high-quality of care and reduction of maternal and neonatal mortality.

### Proposed core indicator

For both planning and monitoring of staffing of maternity and newborn units, an indicator set is needed that addresses the above considerations. The neonatal intensive care community has largely adopted metrics based on shift-wise ratios, often adjusted for acuity/ dependency needs. However, for maternal and routine newborn care an equivalent approach has been elusive and no definitive indicator was identified in the present review. Arising from the findings of this review, in order to overcome the inconsistencies in calculation and reporting of ratios, we propose that the Core indicator should be the *Monthly mean delivery unit staffing ratio*, derived from a staff census of those actually present on duty including all Health Care Professional (HCP) cadres on labour ward including medical staff resident or on-call, and operating theatre staff available for Caesarean delivery, but not including students. Census reports (two or three daily depending on the facility's shift pattern) would be averaged in a one-month period. The final staffing ratio is computed as number of annual births/365/[monthly average shift staff census]. Reflecting the focus on provision of EmONC, the scope includes all staff proving basic newborn care (eg resuscitation following delivery, initiation of skin to skin contact/ suckling, thermal care, administration of vitamin K, cord care) but not special or intensive newborn care, for which existing metrics are available. Box 3 provides a worked example for a hypothetical maternity unit, showing how the core indicator is calculated and how it might be applied when estimating staffing needs against a benchmark. In this example using FIGO norms, the minimum ratio excluding surgical staff is a minimum of 1.71 births per nurse/midwife and an ideal ratio is taken as 1.52.

Box 3 **Worked example: application of the proposed core indicator.**

Operational indicator definition: The number of annual births/365/monthly average shift staff census.

A hypothetical District Hospital in Eastern Africa under Government managing authority provides maternity care to 5,000 mothers, some receiving all their antenatal care at the hospital owing to geographical proximity but two thirds of whom are referred from surrounding Health Centers because of risk factors identified antenatally or for complications in labor. The hospital has a newborn unit in a side ward that is able to provide thermal and nutritional support but has not yet developed a capacity for CPAP or care of newborns below 1500 g, who are typically referred to the nearest University Hospital where possible. There is an operating theatre on site but access is shared with general surgical services. Operative deliveries are undertaken by Clinical Officers or Medical Officers with intermittent availability of a specialist Obstetrician or Family Medicine graduate with surgical training. There is 24 resident anesthesiology and pediatric support at Clinical Officer level with telephone access to advice from specialists. Midwifery and newborn care providers are predominantly Registered Nurse-Midwives, many of whom hold Bachelor-level qualifications but there are also other professional cadres including Nurse Midwife Technicians with certificate-level qualifications. Medical staff typically attend the maternity ward when called but do a hand-over ward round at the start of each shift.

The hospital operates two shifts per 24 h, with a longer night shift starting at 5 pm and a day shift starting at 7 am. The nursing and midwifery staffing complement includes staff employed on permanent Government contracts and posted to the maternity unit, staff on limited-duration contracts and supplemented by ‘locum’ staff providing cover on a casual basis where needed.

The hospital has a team of Matrons whose responsibilities include checking on staff presence on each shift and collating reports for hospital management.

To operationalize the Core Indicator, the respective Matron or her deputy completes a nursing/ midwifery staff census for each shift, with the following template. The form would also contain a box to be ticked to indicate whether or not the complement of staff includes those providing operating theatre nursing care- in this case it does not, as they are counted in the Operating Theatre Department staffing rotas not in the Maternity unit.

**Table:** 

DD/MM/YY	Day or Night Shift (circle as appropriate)	Is this a Weekend day or Holiday (circle as appropriate)
Serial no.	Staff name	*Other details about the staff as needed for additional indicators*

In this example, the collated returns for one calendar month are summarized by averaging the count of staff on day and night shifts.

*November:* Average 5 staff per shift.

Over a year, the monthly averages are averaged to provide the Monthly Average.

*Year 2022*: Average 5.3 staff per shift.

The hospital's maternity register is used to provide data that is entered into DHIS2 (the Health Management Information System) for nationally mandated returns. The number of births for the year is readily available, in this example 5,000, or just under 14 births per day on average.

The Core Indicator ratio is therefore 5,000/365/5.3 = **2.58 births per nurse/midwife**.

Noting the absence of definitive norms and standards, the hospital has decided to test against the FIGO recommended norms. These specify that a unit of this size that provides surgical care should have a **minimum of 8 staff** plus three with surgical experience per shift, and ideally 9 plus four. The above data collection did not include the nurses providing surgical cover as in this particular hospital these are on the staffing complement of the operating theatre and they are not allocated exclusively for maternity cases. The FIGO minimum ratio would therefore be **1.71 births per nurse/midwife**, and the FIGO ideal ratio would be 1.52, so this benchmark is not met in the present case.

The hospital would like to use this approach to calculate how many additional staff they would need to deploy to at least meet the FIGO minimum, as part of their quality improvement plan. They can do this by taking into account their normal work, leave and sickness absence patterns. The Human Resources department has estimated from previous records that a member of the nursing / midwifery staff on a full-time contract is likely to be present and working for 220 out of 365 days each year, allowing for realistic off-duty days, training absences, vacation, sickness, and funeral attendances. A working day is defined nationally as 8 h, so the annual hours anticipated to be actually worked per full time staff in post would be 1,760 h. To achieve the FIGO minimum of 8 staff per shift, it is necessary to cover 8 × 10 × 365 = 29,200 day shift hours and 8 × 14 × 365 = 40,880 night shift hours, total 70,080 h (noting that in this particular hospital there are two shifts per 24 h and the night shift is longer). This would be achieved by employing 70,080 / 1,760 = 39.81 Full Time Equivalent (FTE) staff. The current complement is 5.3 × 10 × 365 = 19,345 day shift hours and 5.3 × 14 × 365 = 27,083 night shift hours, totaling 46,428 h provided by 26.38 FTE staff. The gap to close to meet the minimum benchmark would therefore represent new hires of 39.81–26.38 = **13.43 FTE staff**. The new hires could be a mix of part time or full time staff depending on local conditions as long as the total FTE requirement is met.

## Implications for research

Implementation research is needed to develop and test the practical application of this core indicator in a range of maternity unit settings, its integration into health management information systems such as DHIS-2 and the need for additional related indicators reflecting other essential aspects of care such as QIS and the participation of other clinicians in maternal, newborn and post abortion care. Other than in the field of newborn intensive care, the current literature is not consistent regarding relationships between staffing levels (however measured) and outcomes and research is needed to assess the performance of the above proposed core indicator in this regard, and test its sensitivity to change following adjustment of staffing levels using benchmarks derived from its application.

## Limitations

It should be noted that the present scoping review was not intended to focus specifically on Post Abortion Care although abortion related complications contribute to the burden of maternal mortality and are reflected in the EmONC Signal Functions: this is an important gap for future study.

Limitations of the present scoping review include the limited searching for national Ministry of Health and development partner documents and the restriction to English and French language sources. It is highly likely that informative reports and documents exist in off line formats and in other languages. Data extraction was undertaken by individual investigators rather than the ideal of two independent extractors owing to limitations of time and resources.

## Data Availability

The original contributions presented in the study are included in the article/Supplementary Material, further inquiries can be directed to the corresponding author/s.

## Footnotes

^1^Also known as WTE, Whole Time Equivalent
